# The rs4646 and rs12592697 Polymorphisms in *CYP19A1* Are Associated with Disease Progression among Patients with Breast Cancer from Different Racial/Ethnic Backgrounds

**DOI:** 10.3389/fgene.2016.00211

**Published:** 2016-12-02

**Authors:** Reina Armamento-Villareal, Vallabh O. Shah, Lina E. Aguirre, Angela L. W. Meisner, Clifford Qualls, Melanie E. Royce

**Affiliations:** ^1^Department of Internal Medicine, Baylor College of MedicineHouston, TX, USA; ^2^Department of Internal Medicine, Michael E. DeBakey VA Medical CenterHouston, TX, USA; ^3^Department of Biochemistry and Molecular Biology, University of New Mexico Health Science CenterAlbuquerque, NM, USA; ^4^New Mexico Tumor Registry, University of New Mexico Health Sciences CenterAlbuquerque, NM, USA; ^5^Department of Internal Medicine, New Mexico VA Health Care SystemAlbuquerque, NM, USA; ^6^Department of Mathematics, University of New Mexico Health Science CenterAlbuquerque, NM, USA

**Keywords:** *CYP19A1* polymorphisms, breast cancer, racial disparity, aromatase, women

## Abstract

Given the racial/ethnic disparities in breast cancer, we evaluated the association between *CYP19A1* single nucleotide polymorphisms (SNPs) on disease progression in women with breast cancer from different racial/ethnic backgrounds. This is a cross-sectional analysis of data from 327 women with breast cancer in the Expanded Breast Cancer Registry program of the University of New Mexico. Stored DNA samples were analyzed for *CYP19A1* SNPs using a custom designed microarray panel. Genotype-phenotype correlations were analyzed. Of the 384 SNPs, 2 were associated with clinically significant outcomes, the rs4646 and rs12592697. The T allele for the rs4646 was associated with advanced stage of the disease at the time of presentation (odds ratio [OR]:1.8, confidence intervals [CI]: 1.05–3.13, *p* < 0.05) and a more progressive disease (OR: 2.1 [CI: 1.1–4.0], *p* = 0.04). For the rs12592697, the variant T allele was more frequent in Hispanic women and associated with a more progressive disease (OR: 2.05 [CI: 1.0–4.0], *p* = 0.04). However, further analysis according to menopausal status showed that the association between these 2 SNPs with disease progression or the stage at diagnosis are confined only to postmenopausal women. The odds ratios of disease progression among postmenopausal women carrying the T allele for the rs4646 and rs12592697 are 3.05 (1.21, 7.74, *p* = 0.02) and 3.80 (1.24, 11.6, *p* = 0.02), respectively. Regardless, differences in disease progression among the different genotypes for both SNPs disappeared after adjustment for treatment. In summary, the rs4646 and the rs12592697 SNPs in *CYP19A1* are associated with differences in disease progression in postmenopausal women. However, treatment appears to mitigate the differences in genetic risk.

## Introduction

Breast cancer is the most common cancer diagnosed among women in the US regardless of race or ethnicity; however, disparities in both breast cancer incidence and mortality exist between the various racial/ethnic groups (ACS Cancer Facts & Figures, 2014). The reason for these disparities is likely multifactorial. Data from epidemiological studies suggest that socioeconomic status and educational level, as well as body mass index, may contribute to the difference in prognosis in different racial groups (Berz et al., 2009; Amadou et al., 2014; Shariff-Marco et al., 2014). However, several reports suggest that tumor biology, which accounts for the indolent or aggressive nature of the disease, may explain the disparities in the outcomes in these racial/ethnic groups (Carey et al., 2006; Hines et al., 2011). For instance, results from a study showed higher prevalence of estrogen receptor negative (ER-) tumors and higher proportion of human epidermal growth factor receptor 2 positive (HER2+) tumors in Hispanic women (Hines et al., 2011), while others reported that African-Americans (AA) are more likely to present with ER- than non-Hispanic white (NHW) women (Chen et al., 1994; Chu et al., 2001).

The *CYP19A1* encodes for aromatase, the enzyme responsible for the conversion of the adrenal androgen androstenedione to estrone; the main source of estrogen production in postmenopausal women (Forney et al., 1981). Several polymorphisms of *CYP19A1* were found to be associated with differences in enzyme activity and circulating estradiol levels in postmenopausal women and in elderly men (Gennari et al., 2004; Somner et al., 2004; Riancho et al., 2009). It is assumed that the alteration in hormone levels associated with these polymorphisms is responsible for the observed differences in the risk for breast cancer (Kristensen et al., 2000; Talbott et al., 2008; Sergentanis and Economopoulos, 2010), bone loss and fractures (Masi et al., 2001; Gennari et al., 2004; Somner et al., 2004), and response to aromatase inhibitors (AI) in certain gene variants (Colomer et al., 2008; Garcia-Casado et al., 2010). However, little is known about the influence of genetic variations in *CYP19A1* on the differences in disease behavior among different racial/ethnic groups. Since genetic polymorphisms in the CYP450 genes vary according to race/ethnicity (Napoli et al., 2009), it is possible that polymorphisms in *CYP19A1* may, in part, account for the racial/ethnic differences in the risk and disease behavior of hormone-related diseases such as breast cancer. For instance, it may impact disease progression and response to endocrine therapy for breast cancer that could ultimately translate into racial differences in disease outcomes. The objectives of this study are to determine the prevalence of *CYP19A1* polymorphisms and their association with disease progression among women from different racial/ethnic backgrounds. We hypothesize that certain polymorphisms in *CYP19A1* are associated with racial/ethnic differences in allele frequencies resulting in differences in the risk of disease progression and stage at presentation among women with breast cancer.

## Materials and methods

### Study design

This is an analysis of the clinical data of the patients participating in the Expanded Breast Cancer Registry (EBCR) program at the University of New Mexico Comprehensive Cancer Center (UNMCCC), Albuquerque, NM, USA and who had at least 1 follow-up since enrollment. The study was conducted in accordance with the guidelines in the Declaration of Helsinki for the ethical treatment of human subjects. The protocol was approved by the University of New Mexico Institutional Review Board. The study participants were recruited primarily from the patients attending the UNMCCC Breast Clinic. A written informed consent was obtained from each subject after a detailed explanation of the nature of the study. The EBCR project was initiated in the month of Februray 2006 and follow-up of patients is ongoing. The primay objective is to estblish a registry that will prospectively collect specified tissue samples along with demographics, clinicopathologic variables and treatment information on breast cancer patients seen at the UNMCCC. The plans for the cohort is to augment the existing hospital registry and assist researchers in identifying unique factors that may impact outcomes of patients with breast cancer in New Mexico. Stored DNA samples from patients enrolled in the EBCR were analyzed for polymorphisms in *CYP19A1*, and stored serum samples were assayed for serum estradiol.

### Study population

For this specific study, the population consists of the first 327 women participants of the EBCR program. These participants were followed-up at the UNMCCC, Albuquerque, NM, USA every 6 months for 1 to up to 6 years, from 2/16/2006 to 03/06/2012. Although, males were not specifically excluded, the first 327 participants of the EBCR included in this study did not include any males, likely because breast cancer is less common among men. The specific inclusion and exclusion criteria for the EBCR are described below. This study was not designed to target a subgroup of participants considered as members of the vulnerable population group.

### Inclusion criteria

All patients who are 18 years of age or older with pathological diagnosis of breast cancer; regardless of gender, ethnicity/race, stage of disease or treatment; must be less than or equal to 1 year from the diagnosis of non-metastatic breast cancer, or less than or equal to 1 year from initiation of treatment for metastatic disease; must be available for clinical follow-up; and must be willing to provide written, informed consent.

### Exclusion criteria

Patients who are younger than 18 years of age; without pathological proof of diagnosis of breast cancer; unable to provide clinical follow-up; unable to provide informed consent in accordance with institutional and federal guidelines.

### Data collection

The clinical chart of these subjects were examined primarily for disease progression, stage and treatment. A progressive disease is defined as transformation to a more advanced stage compared to the stage at the time of presentation, recurrent disease or the development of a new tumor. Other data collected include self-reported race/ethnicity, estrogen receptor/progesterone receptor (ER/PR) and HER2 status, age at diagnosis, menopausal status, years since menopause, body mass index (BMI) which was calculated as weight in kilograms/square of the height in meters, and family history of breast cancer.

### Biochemical analysis

Baseline serum estrodiol assay was performed from stored serum using ultrasensitive Elisa assay (Alpco Diagnostics, Salem, NH). The assay sensitivity for estradiol using this kit was 1 pg/ml. The coefficient of variablity of this assay in our lab is <10% (Aguirre et al., 2015).

### Genotyping

Genotyping was performed by oligonucleotide microarray (BeadArray technology, Golden Gate [GG]; Illumina). DNA probes—Custom genotyping panel was created for *CYP19A1* using Illumina's® Golden Gate® technology and Assay Design Tool (ADT). SNP's were selected within the *CYP19A1* and submitted to Illumina® for probe design using Illumina's® Assay Design Tool (ADT). QC metrics are returned and SNPs were selected based on their likelihood for success within the parameters of the assay and their potential to be functionally active according to their location in the gene, or their previous associations with differences in estradiol levels, gene expression, transcriptional acitivity, and reported risk for breast cancer.

At the time of conception of this study, ~980 SNPs were listed in the NCBI. We selected panel of 384 validated SNPs that met the above criteria.

SNPs were removed from the analysis if they had call rates of <94%, with minor allele frequency of <5% and deviated from the Hardy-Weinberg equilibrium, leaving 119 SNPs available for analysis in our current study. In this study, the main reason SNPs were excluded was for low minor allele frequency. Regardless, we paid particular attention to the SNPs reported to be associated with breast cancer outcomes (Hadfield and Newman, 2012); (rs4646, rs10046, rs700519, rs700518) (Colomer et al., 2008; Garcia-Casado et al., 2010; Hadfield and Newman, 2012); and SNPs associated with differences in circulating estradiol levels (rs700518 and rs1062033, rs10046; Dunning et al., 2004; Riancho et al., 2007, 2009), ezymatic activity (rs1062033) (Riancho et al., 2009) and gene expression (rs10046) (Kristensen et al., 2000; Colomer et al., 2008; Garcia-Casado et al., 2010; Hadfield and Newman, 2012).

### Statistical analysis

Results were expressed as means ± *SD*. The primary outcome in this study is disease progression, and disease stage at the time of presentation or diagnosis is the secondary outcome. We also analyzed other variables including estradiol levels, hormone receptor and HER2 receptor status which may contribute to disease (breast cancer) behavior. Group comparisons were done by analysis of variance (ANOVA) for continuous variables and by chi-square analysis for categorical variables. Odds ratios were calculated using logistic regression analysis. Kaplan-Meier curves were obtained by survival analysis. The data were managed using Excel 2010 (Microsoft, Redmond, WA) and were analyzed using SAS version 9.3 (SAS Institute, Inc., Cary, NC, USA).

## Results

Data from 327 women were analyzed. Table 1 shows the clinical characteristics of the women. There were 325 women who responded to their racial/ethnic background and 322 women whose menopausal status can be verified or inferred. For the purpose of the study, women who did not respond to the menopausal status questionnaire, were considered menopausal if over the age of 50, while those who continued have periods after age 50 were considered to be perimenopausal. As shown in the table, majority of our subjects were NHW (50.5%) and Hispanic (36.6%) women. As expected more women had HR+ disease and significantly less HER2+ tumors. Only 18.7% of women had triple negative breast cancer (TNBC). Majority of the women (76.2%) presented at early stages of the disease (stage 0–II) while 23.8% presented at later stages of the disease (Stage III–IV). Over the observation period of 6 years, 57 of the 312 patients (18.3%) had progressive disease. Of the 312 women, 195 were on antiestrogen therapy namely: 83 were on aromatase inhibitors, 95 were on selective estrogen receptor modulators (SERMs), while 17 were initially on either a SERM and switched to an AI or vice-versa.

**Table 1:** **Clinical characteristics of the entire study population**.

Age (years)	53.2 ± 10.79
BMI (kg/m^2^)	28.6 ± 6.68
Menopausal status	(*N* = 322)
Premenopausal	105 (32.6%)
Perimenopausal	27 (8.4%)
Postmenopausal	190 (59.0%)
History of postmenopausal hormone therapy	103/324 (31.8%)
Racial/Ethnic distribution	(*N* = 325)
Non-Hispanic whites	164 (50.5%)
Hispanics	119 (36.6%)
African-Americans	11 (3.4%)
American Indians	18 (5.5%)
Asians	2 (0.6%)
Multiracial	11 (3.4%)
Baseline serum estradiol (pg/ml)	24.5 ± 28.4
Receptor status	
ER	
Positive	224/312 (71.8%)
Negative	88/312 (28.2%)
PR	
Positive	180/308 (58.4%)
Negative	128/308 (41.6%)
HER2	
Positive	64/287 (22.3%)
Negative	223/287 (77.7%)
Triple negative	59/315 (18.7%)
Stage at presentation (*N* = 324)	
Stage 0–II	247 (76.2%)
Stage III–IV	77 (23.8%)
Progressive disease	57/312 (18.3%)
Baseline serum estradiol	24.5 ± 28.4

*Formats of cell entries are mean ± SD or frequency/n (%). BMI, body mass index; ER, Estrogen receptor, PR, progesterone receptor; HER2, Human epidermal growth factor receptor 2*.

Table 2 shows the comparison beween NHW and Hispanic women which constitute the majority of the racial groups in our population. Hispanic women were much younger and have higher BMI than the NHW. There were also significantly more Hispanic women diagnosed with breast cancer during the premenopausal stage compared to NHW, while significanlty more NHW women had a history of postmenopausal hormone replacement therapy (HRT). There was no difference in the number of HR+ tumors in both NHW and Hispanic women. However, there were signficantly more NHW women who were HER2+. Hispanic women tend to have a higher proportion of TNBC and more progressive disease but the differences were not statistically signficant.

**Table 2:** **Comparison between non-Hispanic and Hispanic women participants**.

	**Non-Hispanic white (*n* = 164)**	**Hispanic (*n* = 119)**	***P*-values**
Age	55.5 ± 10.1	50.7 ± 10.9	**0.001**
BMI	27.4 ± 6.6	29.9 ± 6.3	**0.002**
Menopausal status	(*N* = 149)	(*N* = 101)	**0.01**
Premenopausal	34 (22.82%)	41 (40.6%)	
Perimenopausal	9 (6.04%)	4 (4.0%)	
Menopausal	106 (71.14%)	56 (54.4%)	
History of postmenopausal hormone therapy	70/163 (42.9%)	18/118 (15.3%)	<**0.001**
Receptor status			
ER	(*N* = 157)	(*N* = 114)	0.96
Positive	112/157 (71.3%)	81/114 (71%)	
Negative	45/157 (28.7%)	33/114 (29%)	
PR	(*N* = 158)	(*N* = 108)	0.67
Positive	88/158 (55.7%)	63/108 (58.3%)	
Negative	70/158 (44.3%)	45/108 (41.7%)	
HER2	(*N* = 145)	(*N* = 104)	<**0.05**
Positive	39/145 (26.9%)	17/104 (16.4%)	
Negative	106/145 (73.1%)	87/104 (83.6%)	
Triple negative	26/158 (16.5%)	26/115 (22.6%)	0.20
Progressive disease	24/157 (15.3%)	25/113 (22.1%)	0.15
Stage at diagnosis			0.65
Stage 0–II	125/163 (76.7%)	87/117 (74.4%)	
III–IV	38/163 (23.3%)	30/117 (25.6%)	
Baseline serum estradiol	25.1 ± 23.5	27.6 ± 36.4	0.66

*Formats of cell entries are mean ± SD or frequency/n (%). BMI, body mass index; ER, Estrogen receptor; PR, progesterone receptor; HER2, Human epidermal growth factor receptor 2. Bold values indicate that the p-value is statistically significant*.

We found two SNPs that are associated with important clinical outcomes, the rs4646 located at the 3′UTR and the rs12592697 located in the intronic sequence. Three hundred seven subjects were successfully genotyped for the rs4646 polymorphism with the following genotype frequency: CC = 145, CT = 128 and TT = 34. As shown in Table 3, the minor T allele (the variant allele) for this SNP was associated with a more advanced disease stage at the time of diagnosis (odds ratio [OR]:1.8, confidence intervals [CI]: 1.05–3.13, *p* < 0.05). In addition, the minor T allele was also associated with a more progressive disease over the observation period (OR: 2.1 [CI: 1.1−4.0], *p* = 0.04). However, the difference in disease behavior between the genotypes was lost when adjusted for treatment (*p* = 0.13) status. There was no difference in the proportion of HR+ and HER2+ tumors and TNBC between the genotypes. There was no significant difference in genotype frequency for this polymorphism across racial groups; in particular, there was no difference in frequency between the NHW (CC = 77/154, CT = 63/154, TT = 14/154 and Hispanic women (CC = 50/112, CT = 47/112, TT = 15/112).

**Table 3:** **Clinical features and tumor characteristics of patients according to the rs4646 polymorphism**.

	**CC (*N* = 145)**	**CT/TT (*N* = 162)**	***P***
Age	53.7 ± 10.5	52.5 ± 11.0	0.33
BMI	28.5 ± 6.8	29.0 ± 6.8	0.52
Menopausal status	(*N* = 144)	(*N* = 159)	0.89
Premenopausal	47 (32.6%)	55 (35.6%)	
Perimenopausal	9 (6.3%)	11 (6.9%)	
Postmenopausal	88 (61.1%)	93 (58.5%)	
History of postmenopausal hormone therapy	47/142 (33.1%)	49/162 (30.2%)	0.59
Racial/Ethnic distribution			
Non-Hispanic whites (*N* = 154)	77/154 (50.0%)	77/154 (50.0%)	0.62
Hispanic whites (*n* = 112)	50/112 (44.6%)	62/112 (55.4%)	
^*^Others (*N* = 41)	18/41 (43.9%)	23/41 (56.1%)	
Receptor Status			
ER	(*N* = 141)	(*N* = 153)	0.96
Positive	101/141 (71.6%)	110/153 (71.9%)	
Negative	40/141 (28.4%)	43/153 (28.1%)	
PR	(*N* = 137)	(*N* = 153)	0.37
Positive	85/137 (62.0%)	87/153 (56.9%)	
Negative	52/137 (38.0%)	66/153 (43.1%)	
HER2	(*N* = 130)	(*N* = 142)	0.62
Positive	27/130 (20.8%)	33/142 (23.2%)	
Negative	103/130 (79.2%)	109/142 ((76.8%)	
Triple negative	26/141 (18.4%)	30/156 (19.2%)	0.86
Progressive disease	17/137 (12.4%)	36/155 (23.2%)	**0.02**
Stage at diagnosis			
Stage 0–II	118/144 (81.9%)	113/160 (70.6%)	**0.02**
III–IV	26/144 (18.1%)	47/160 (29.4%)	
Estradiol (pg/ml)	24.1 ± 32.1	26.0 ± 24.4	0.71

Formats of cell entries are mean ± SD or frequency/n (%). BMI, body mass index;

* *African-Americans, Native Americans, Asians, and multiracial. ER, Estrogen receptor; PR, progesterone receptor; HER2, Human epidermal growth factor receptor 2. Bold values indicate that the p-value is statistically significant*.

Another SNP found to be associated with a difference in clinical outcome was the rs12592697 (Table 4). Three hundred six subjects were successfully genotyped for this polymorphism; the genotype frequency was CC = 111, CT = 151, and TT = 44. There were proportionately greater number of women with the minor T allele who were premenopausal compared to those without the T allele. Among the 293 patients with known ER status, significantly more patients classified as ER- were carriers of the T allele. There was a trend for women with PR- tumors to be carriers of the T allele. In addition, among patients with data on disease progression, a greater proportion of subjects carrying the T allele (22%) had a progressive disease compared to 12.0% of patients without the T allele (OR: 2.05 [CI 1.0–4.0), *p* = 0.04]. Further analysis showed that ER positivity or PR positivity are related to progression for this SNP (both *P* < 0.001). However, the combined ER positivity and PR positivity is not related to progression among variants of rs12596297 (*P* = 0.75). Again, the difference in disease behavior disappeared after adjusting for treatment status (*p* = 0.26). Among NHW (*n* = 154), the genotype frequency was: CC = 64, CT = 70, and TT = 20, while in Hispanics (*n* = 111) it was: CC = 32, CT = 60, and TT = 19.

**Table 4:** **Clinical features and tumor characteristics of patients according to the rs12592697 polymorphism**.

	**CC (*N* = 111)**	**CT/TT (*N* = 195)**	***P***
Age	53.2 ± 10.4	53.0 ± 11.1	0.90
BMI	28.2 ± 6.9	29.0 ± 6.7	0.36
Menopausal status (*n* = 302)			
Premenopausal	31 (28.4%)	70 (36.3%)	**0.04**
Perimenopausal	12 (11.0%)	8 (4.1%)	
Postmenopausal	66 (60.6%)	115 (59.6%)	
History of postmenopausal hormone therapy	35/108 (32.4%)	62/197 (31.5%)	0.87
Racial/Ethnic distribution			
Non-Hispanic whites (*N* = 154)	64/154 (41.6%)	90/154 (59.6%)	0.10
Hispanic whites (*N* = 111)	32/111 (28.8%)	79/111 (71.2%)	^**^NHW vs. Hispanics only ***P* = 0.03**
^*^Others (*N* = 41)	15/41 (36.6%)	26/41 (63.4%)	
Receptor status			
ER	(*N* = 108)	(*N* = 185)	0.04
Positive	85/108 (78.7%)	125/185 (67.6%)	
Negative	23/108 (21.3%)	60/185 (32.4%)	
PR	(*N* = 137)	(*N* = 182)	0.08
Positive	71/137 (66.4%)	100/182 (54.9%)	
Negative	36/137 (33.6%)	82/182 (45.1%)	
HER2	(*N* = 94)	(*N* = 177)	0.50
Positive	23/94 (24.5%)	37/177 (20.9%)	
Negative	71/94 (75.5%)	140/177 (79.1%)	
Triple negative	19/107 (17.8%)	38/191 (19.9%)	0.65
Progressive disease	13/108 (12.0%)	40/182 (22.0%)	**0.03**
Stage at diagnosis (*n* = 303)			0.49
Stage 0–II	86/110 (78.2%)	144/193 (74.6%)	
III–IV	24/110 (21.8%)	49/193 (25.4%)	
Estradiol (pg/ml)	26.8 ± 37.3	24.3 ± 22.5	0.65

Formats of cell entries are mean ± SD or frequency/n (%), BMI, body mass index;

* African-Americans, Native Americans, Asians, and multiracial. ER, Estrogen receptor; PR, progesterone receptor; HER2, Human epidermal growth factor receptor 2;

** *Comparison of allele/genotype frequency between NHW and Hispanics only by T-test. Bold values indicate that the p-value is statistically significant*.

Although there was no difference in genotype frequency among the different races, a separate comparison between NHW and Hispanics for the rs12596297 showed that the T allele was significantly more prevalent in Hispanic (71.2%) compared to NHW (59.6 %) women (*p* = 0.03).

Since less than 10% of our entire study population consisted of perimenopausal women, we focused our analysis primarily the premenopausal and postmenopausal women. Dividing the appropriate subjects into these two groups showed that the association between the SNPs and outcomes are confined to postmenopausal women only (Tables 5, 6). There were no differences in any outcome measure for rs4646 and rs12592697 among premenopuasal women. By contrast, there were significantly more postmenopausal women carrying the T allele for the rs4646 who have progressive disease and a more advanced stage at the time of diagnosis. For rs12592697, significantly more postmenopausal women carrying the T allele are ER- and have a more progressive disease. In addition, there were trends for carriers of the T allele to have HR- tumors (i.e., less of either ER+, PR+ or both ER+/PR+) compared to those homozygous for the C allele among postmenopausal women. Again, adjustment for treatment status eliminated the differences in disease progression among the genotypes for both SNPs (*p* = 0.13 for rs4646 and *p* = 0.28 for rs12592697).

**Table 5:** **Analysis of the different clinical characteristics and outcomes according to rs4646 and rs12592697 polymorphisms in the ***CYP19A1*** in premenopausal women with breast cancer**.

**rs4646**	**CC (*N* = 85)**	**CT/TT (*N* = 86)**	
ER			
Positive	27/44 (61.4%)	34/53 (64.2%)	0.78
Negative	17/44 (38.6%)	19/53 (35.8%)	
PR			
Positive	25/44 (56.8%)	30/53 (56.6%)	0.98
Negative	19/44 (43.2%)	23/53 (43.4%)	
ER+/PR+	25/43 (58.1%)	30/51 (58.8%)	0.96
HER2+	9/41 (21.9%)	11/50 (22%)	0.99
Triple negative	12/44 (27.3%)	17/53 (32.1%)	0.61
Progressive disease	9/44 (20.5%)	15/53 (28.3%)	0.37
Stage at diagnosis			
Stage 0–II	35/46 (76.1%)	36/55 (65.5%)	0.24
III–IV	11/46 (23.9%)	19/55 (34.5%)	
Estradiol (pg/ml)	36.2 ± 26.2	37.8 ± 44.2	0.89
**rs12592697**	**CC**	**CC/TT**	
ER			
Positive	22/31 (71.0%)	39/66 (59.1%)	0.26
Negative	9/31 (29.0%)	27/66 (40.9%)	
PR			
Positive	19/30 (63.3%)	36/67 (53.4%)	0.38
Negative	11 (36.7%)	31/67 (46.3%)	
ER+/PR+	19/30 (63.3%)	36/65 (55.4%)	0.47
HER2+	5/26 (19.2%)	15/65 (23.1%)	0.69
Triple negative	9/31 (29.0%)	20/66 (30.3%)	0.90
Progressive disease	8/31 (25.8%)	16/66 (24.2%)	0.87
Stage at diagnosis (*n* = 303)			
Stage 0–II	20/30 (66.7%)	51/70 (72.9%)	0.40
III–IV	11/31 (35.5%)	19/70 (27.1%)	
Estradiol (pg/ml)	47.2 ± 61.1	33.6 ± 24.4	0.30

*Formats of cell entries are mean ± SD or frequency/n (%), ER+, Estrogen receptor positive; PR+, progesterone receptor positive; HER2, Human epidermal growth factor receptor 2*.

**Table 6:** **Analysis of the different clinical characteristics and outcomes according to rs4646 and rs12592697 polymorphisms in the ***CYP19A1*** in postmenopausal women with breast cancer**.

**rs4646**	**CC *N* = 85 (49.7%)**	**CT/TT *N* = 86 (50.3%)**	
ER			
Positive	68/87 (71.2%)	64/87 (73.6%)	0.48
Negative	19/87 (21.8%)	23/87 (26.4%)	
PR			
Positive	54/82 (65.9%)	48/86 (55.8%)	0.18
Negative	28/82 (34.1%)	38/86 (44.2%)	
ER+/PR+	53/82 (64.6%)	48/84 (57.1%)	0.32
HER2	17/80 (21.3%)	19/79 (24.1%)	0.67
Triple negative	11/85 (12.9%)	13/87 (14.9%)	0.71
Progressive disease	7/81 (8.6%)	20/88 (22.7%)	**0.01**
Stage at diagnosis			
Stage 0–II	72/86 (83.7%)	65/91 (71.4%)	**0.05**
III–IV	14/86 (16.3%)	26/91 (28.6%)	
Estradiol pg/ml	13.6 ± 13.0	15.8 ± 16.1	0.54
**rs12592697**	**CC**	**CC/TT**	
ER			
Positive	52/62 (83.9%)	80/113 (70.8%)	**0.05**
Negative	10/62 (16.1%)	33/113 (29.2%)	
PR			
Positive	42/61 (68.9%)	60/108 (55.6%)	0.09
Negative	19/61 (31.1%)	48/108 (44.4%)	
ER+/PR+	42/60 (70.0%)	59/107 (55.1%)	0.06
HER2	15/55 (27.3%)	21/105 (20.0%)	0.30
Triple negative	7/60 (11.7%)	18/113 (15.9%)	0.45
Progressive disease	4/62 (6.5%)	23/108 (21.3%)	**0.01**
Stage at diagnosis (*n* = 303)			
Stage 0–II	52/63 (82.5%)	86/115 (74.8%)	0.24
III–IV	11/63 (17.5%)	29/115 (25.2%)	
Estradiol pg/ml	14.4 ± 14.5	14.6 ± 14.6	0.95

*Formats of cell entries are mean ± SD or frequency/n (%), ER+, Estrogen receptor positive; PR+, progesterone receptor positive; HER2, Human epidermal growth factor receptor 2. Bold values indicate that the p-value is statistically significant*.

**Figure 1:**
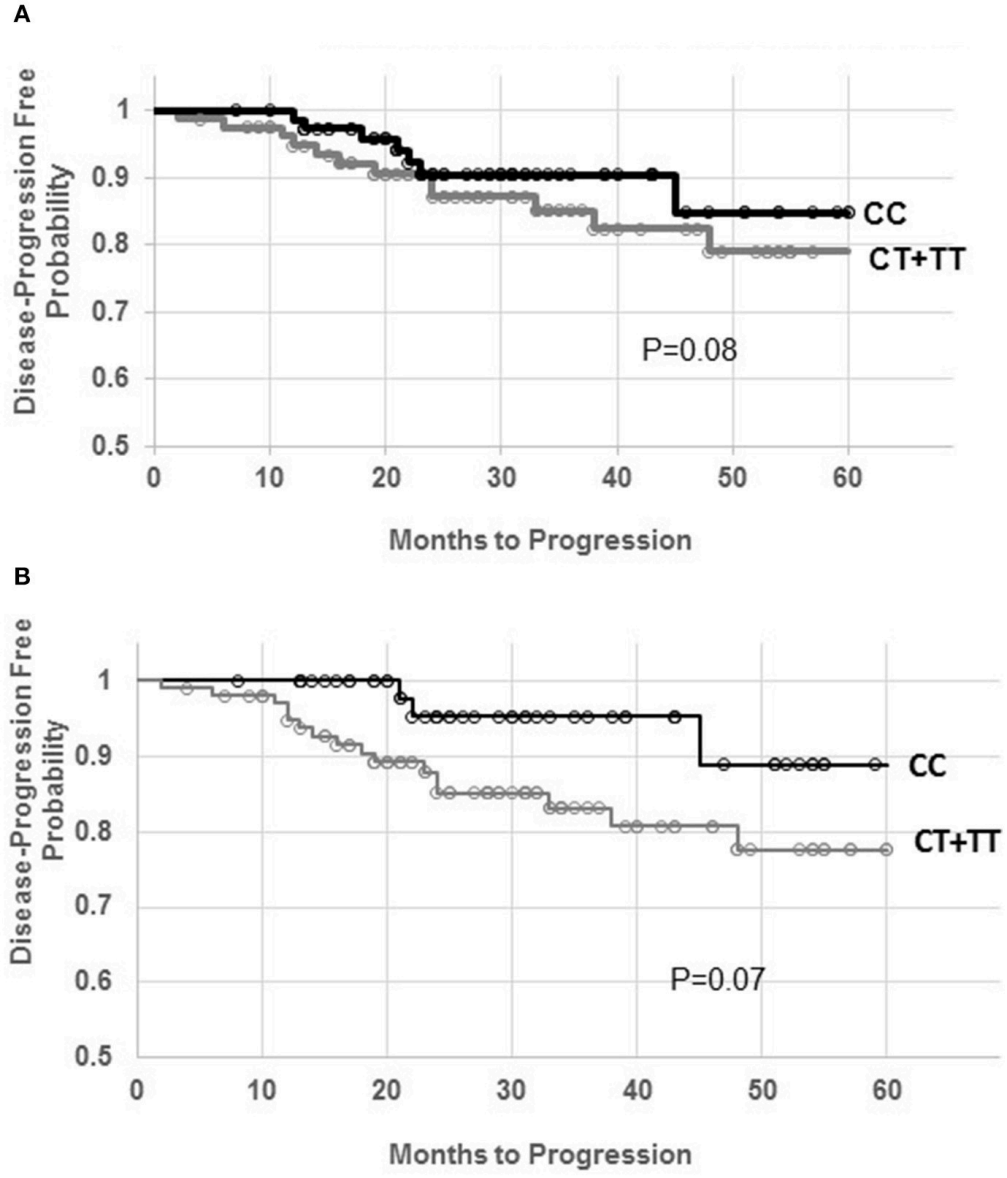
**Kaplan-Meier distribution for disease progression-free probability among the genotypes for the rs4646 (A)** and rs12592697 **(B)** polymorphisms in the *CYP19A1*. Circles represent time of data censor.

Time to progression using Kaplan-Meier analyses among postmenopausal women showed a trend for a lower disease progression-free probabality among women carrying the T allele for both SNPs, Figure 1 (Figure 1A for rs4646 and Figure 1B for rs12592697). The odds ratios of disease progression among postmenopausal women carrying the T allele for the rs4646 and rs12592697 are 3.05 (1.21, 7.74, *p* = 0.02) and 3.80 (1.24, 11.6, *p* = 0.02), respectively. For untreated postmenopausal women, the odds ratios of disease progression for carriers of the T allele of the rs4646 and rs12592697 are 7.49 (1.90, 29.5, *p* = 0.004) and 3.01 (0.77, 11.7, *p* = 0.11), respectively. Analysis of women on AI alone (*n* = 83) show odds ratios for disease progression for carriers of the T allele for the rs4646 and rs12592697 to be 1.22 (0.36, 4.18 *p* = 0.75) and 3.18 (0.65, 15.7 *p* = 0.15), respectively.

The genotype frequencies for both rs4646 and rs12592697 were in Hardy-Weinberg equilibrium in our population (http://www.had2know.com/academics/hardy-weinberg-equilibrium-calculator-2-alleles.html).

## Discussion

Our results demonstrated that rs4646 polymophism of *CYP19A1* was associated with differences in the disease progression and stage at the time of diagnosis. The T allele was associated with a more advanced stage of the disease at the time of presentation and a more progressive disease. We did not find any ethnic differences in genotype frequencies for the rs4646 polymorphism. However, the rs12592697 polymorphism, was associated with ethnic differences in genotype frequency; there were significantly higher proportion of Hispanic white women who were carriers of the minor T allele relative to NHW women. In addition, more women with progressive disease were carriers of the T allele. A separate analysis between premenopausal and postmenopausal women showed that the associations between the 2 SNPs and disease progression were only observed in postmenopausal women. Similarly, the association between rs4646 and the disease stage at the time of diagnosis was only observed in postmenopausal women. However, it appeared that the influence of both polymorphism on disease progression was attenuated by treatment status.

In postmenopausal women, most of the circulating estrogen comes from the conversion of adrenal androgens to estrone by the enzyme aromatase, which is in turn interconverted to estradiol. Several polymorphisms in the aromatase or *CYP19A1* have been reported to be associated with hormone-related disease such as osteoporosis and breast cancer (Masi et al., 2001; Somner et al., 2004; Talbott et al., 2008; Sergentanis and Economopoulos, 2010). The rs4646 polymorphism (C to T) at the 3′UTR has been reported to be associated with differences in disease-free survival. For instance, the variant T allele was found to be part of a haplotype that is associated with poor disease-free survival in Taiwanese women (Kuo et al., 2013). Furthermore, in a treatment study, the same allele was associated with a lower progression-free survival compared to those homozygous for the wild type (C) allele treated with letrozole given as a neoadjuvant treatment in postmenopausal women of Spanish ancestry with stage II–III ER/PR (+) breast cancer (Garcia-Casado et al., 2010). By contrast, in a study among Chinese women with metastatic breast cancer, patients with the variant allele had statistically significantly longer overall survival compared to women homozygous for the wild type (37.3 vs. 31.6 mos, *p* = 0.007; Liu et al., 2013). Likewise, another study in postmenopausal women with advanced breast cancer of Spanish ancestry showed a significant improvement in time to progression among those with the variant T allele (17.2 vs. 6.4 mos; *p* = 0.02) treated with letrozole (Colomer et al., 2008). Our study showed that patients with the T allele for the rs4646 presented with a more advanced disease than those without the T allele mostly in postmenopausal women. Moreover, these patients also had a more progressive disease. Although, these results seem inconsistent, these studies were conducted in different populations, with different ethnic backgrounds and living in different geographical locations with different dietary and lifestyle habits, all of which may interact with genetics to influence phenotypes.

Polymorphisms in *CYP19A1* vary across racial and ethnic backgrounds (Janicki et al., 2013), and may explain the different susceptibility, disease behavior and response to therapy among hormone-related disorders across racial/ethnic groups. In this study we did not find a racial/ethnic difference in genotype frequency for the rs4646 polymorphism. However, the rs12592697 in the intronic region of *CYP19A1* was associated with differences in genotype frequency between NHW and Hispanic women. To our knowledge, there has been no clinical association reported for this SNP except that it is in linkage with a SNP that maybe associated with significant differences in estradiol levels among the variants (Prescott et al., 2012). In our study, the variant T allele which is more prevalent in Hispanic women, was associated with a higher proportion of ER- tumors and a more progressive course of breast cancer in postmenopausal women. Nonetheless, it remains unknown whether this allele contributes to poorer outcomes of breast cancer among Hispanics relative to NHW women in general.

A variant allele for another polymorphism, the rs10046, has been reported to be associated with higher estradiol levels (Dunning et al., 2004) and higher frequency in women with breast cancer (Kristensen et al., 2000). In our study there was no significant increase in estrogen levels in the variants for both rs4646 and rs12592697 at baseline regardless of menopausal status. However, this finding is not unusual as other studies also failed to show differencess in levels of circulating estrogen in other clinically important *CYP19A1* SNPs (Napoli et al., 2013). Of importance in breast cancer, are data by some investigators of an increase in local aromatase activity in breast tissues in certain variants which may influence hormone levels on the tumor microenvironment and in turn correlate with disease behavior (Kristensen et al., 2000). In addition, prior investigators have reported a higher risk for breast cancer among women with higher levels of androgens independent of estrogen levels (Missmer et al., 2004; Peters et al., 2009) and although androgen receptor positivity is associated with better prognosis in ER+ breast cancers (Peters et al., 2009), it is also associated with acquired resistance to anti-estrogen (De Amicis et al., 2010; Rechoum et al., 2014). Whether androgen levels and androgen receptor activity have roles as mediators in the observed association between the SNPs and outcomes have not been explored in our study.

Aside from being considered as a risk factor for breast cancer, a high body weight is also associated with higher risk for recurrence and reduced survival (Jain et al., 2013; Azrad and Demark-Wahnefried, 2014). In addition, there are studies suggesting that AIs may not be as effective in obese women with HR+ breast cancer as compared to women with normal weight (Pfeiler et al., 2013). Since the prevalence of obesity is higher in certain racial/ethnic groups (An, 2014), it is possible that this may contribute to poor breast cancer outcomes in certain racial/ethnic groups compared to others. In addition, age is an important determinant of prognosis. Younger women who tend to be premenopausal with a higher rate of triple negative tumors at the time of breast cancer diagnosis are likely to have poorer outcomes than older women (Ray and Polite, 2010). In our study, regardless of the findings that Hispanic women had higher BMI, were significantly younger with a greater proportion of them being premenopausal, we cound not find a difference in the risk for a progressive disease between the Hispanic and NHW women in the population as a whole. On the other hand, HER2 positivity which is associated with poorer prognosis is much higher in NHW than Hispanic women and may have modified the effects of age, menopausal status and BMI on disease behavior between the races. However, our sample size is quite limited to make a definitive conclusion. There was no difference in BMI, age and receptor status among genotypes for the rs4646 to account for the difference in disease stage at the time of presentation nor disease progression. Meanwhile, a significant number of women with the T allele for the rs12592697 are premenopausal and are also ER negative. It is possible that these features may have contributed to a more progressive disease among patients with the T allele for this polymorphism. However, analysis by menopausal status showed that the association between these SNPs and our outcomes are only obeserved in postmenopausal women and the association between the 2 SNPs and disease behavior does not exist in premenopausal women. This finding underscores the important role of the aromatase enzyme activity as determinant of hormonal status after cessation of ovarian function. Our findings also suggest that ER positivity or PR positivity may contribute to the association between *CYP19A1* SNPs and outcomes in postmenopausal women, in particular rs12592697, perhaps suggesting an interaction between hormone receptor signaling in some of these SNPs.

Our results showed that the association between the 2 SNPs disappeared with adjustment for treatment. We also found that the T allele in both SNPs was associated with overall higher odds of disease progression mostly in the untreated group, while a separate analysis of women on AIs alone revealed no significant differences in disease progression associated with both SNPs. It is likely that the amount of estrogen present in the postmenopausal period, albeit small, is still able to produce significant difference in biological effects on the breast among women belonging to the different genotypes in the absence of treatment; and this difference is eliminated by antiestrogens resulting in comparable disease progression. On the other hand, it is also conceivable that the limited sample size prevented us from detecting a difference in response, more specifically in the small number of women who are on AI. In addition, as these data are obtained from chart review, the actual compliance (which could affect disease behavior) among these patients on the prescribed therapy remains unknown.

Our study has limitations. Being cross-sectional in design, the data presented here is limited to what is avilable in the patient's medical record. In addition, race/ethnicity was categorized based on self-reporting by the patient (i.e., not verified for instance by ancestry informed markers), and all recruited in the same institution both of which may introduce bias. We also did not correct for multiple comparisons in our study. Furthermore, the relativley short period of follow-up limits our ability to assess the more important outcome which is mortality. Functionality of the SNPs studied is likewise not addressed in this study. Although, the underlying mechanism by which these SNPs influence disease behavior or even response to AI treatment is unclear, it is conceivable that our findings could result in hypothesis generating projects to further elucidate the mechanism for our observations. For instance, it is possible that variant alleles result in enhanced gene expression; or posttranslational modification with change in protein structure resulting in a more active enzyme, or that they could be in linkage with functionally active SNPs resulting in higher estrogenic environment.

## Conclusions

Two SNPs in *CYP19A1* were associated with disease outcomes particularly in the cohort of postmenopausal women diagnosed with breast cancer at our institution. Postmenopausal women with the T allele for the rs4646 polymorphism located at the 3′UTR had a more advanced disease at the time of presentation and a more progressive disease than those without the T allele. Another SNP, rs12592697, in the intronic region of *CYP19A1* was associated with differences in allele frequency between NHW and Hispanic women with breast cancer. The variant T allele was found to be more frequent in Hispanic than NHW women. Furthermore, this allele was associated with a more progressive disease in postmenopausal women relative to the wild type. Despite the genetic predisposition for a more advanced and progressive disease among certain variants for these 2 polymomorphisms, our results suggest that this association is modified by treatment. Specifically, in our patient population, anti-estrogen therapy prevented disease progression among postmenopausal women with risk alleles for *CYP19A1*. If proven to be true, our findings may have significant clinical implications for patients carrying these risk alleles. For instance, in patients harboring the common genetic variations in *CYP19A1*, this information may motivate them to become adherent to their prescribed adjuvant endocrine therapy. It is well documented that in women who start adjuvant endocrine therapy, rates of non-persistence (early discontinuation of medication) range from 20% to 50% and rates of non-compliance (nonconformity to prescribed dosing) range between 16 and 26%, and worsen over time (Hershman et al., 2010). These individualized information may be a useful reminder that could encourage at risk patients to continue with their recommended treatment. This is especially important in light of the updated recommendations for longer adjuvant endocrine therapy in HR+ disease (Burstein et al., 2016) and potential for dramatic increased non-adherence rates to adjuvant endocrine therapy.

## Clinical trials

ClinicalTrials.govs Identifier: NCT00322894 (https://clinicaltrials.gov/ct2/show/NCT00322894?term=new+mexico+breast+cancer+registry&rank=1).

## Author contributions

RA, MR, and VS conceived and designed the experiments; RA, LA, and VS performed the experiments; RA and CQ analyzed the data; RA, AM, and MR contributed reagents/materials/analysis tools; RA, MR, CQ, VS, LA, and AM wrote the paper. Authorship must be limited to those who have contributed substantially to the work reported.

## Funding

This work was supported by NM-SB 532 (Institutional Grant No. 3R62E). VS was supported by the funding from the National Center for Research Resources (5P20RR016480-12) and the National Institute of General Medical Sciences (8P20GM103451-12) of the NIH.

### Conflict of interest statement

The authors declare that the research was conducted in the absence of any commercial or financial relationships that could be construed as a potential conflict of interest.
